# Controls on the Barium and Strontium Isotopic Records of Water Chemistry Preserved in Freshwater Bivalve Shells

**DOI:** 10.1021/acs.est.4c05652

**Published:** 2024-08-30

**Authors:** Kristi S. Dobra, Rosemary C. Capo, Brian W. Stewart, Wendell R. Haag

**Affiliations:** † Department of Geology and Environmental Science, University of Pittsburgh, Pittsburgh, Pennsylvania 15260, United States; ‡ US Forest Service, Southern Research Station, Center for Bottomland Hardwoods Research, Frankfort, Kentucky 40601, United States

**Keywords:** mussel, trace metals, unionid, biomineral, aragonite, fractionation, invasive species, zebra mussel

## Abstract

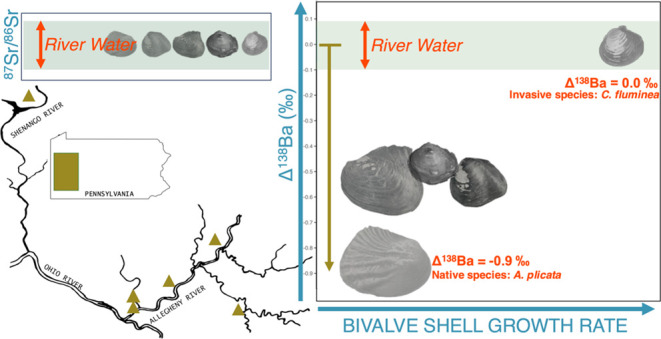

Biogenic carbonates, including bivalve shells, record past environmental conditions, but their interpretation requires understanding environmental and biological factors that affect trace metal uptake. We examined stable barium (δ^138^Ba) and radiogenic strontium (^87^Sr/^86^Sr) isotope ratios in the aragonite shells of four native freshwater mussel species and two invasive species in five streams and assessed the effects of species identity, growth rate, and river water chemistry on shell isotopic composition. Shells were robust proxies for Sr, accurately reflecting ^87^Sr/^86^Sr ratios of river water, regardless of species or growth rate. In contrast, shell δ^138^Ba values, apart from invasive *Corbicula fluminea*, departed widely from those of river water and varied according to species and growth rate. Apparent fractionation between river water and the shell (Δ^138^Ba_shell-water_) reached −0.86‰, the greatest offset observed for carbonate minerals. The shell deposited during slow growth periods was more enriched in lighter Ba isotopes than the rapidly deposited shell; thus, this phenomenon cannot be explained by aragonite precipitation kinetics. Instead, biological ion transport processes linked to growth rate may be largely responsible for Ba isotope variation. Our results provide information necessary to interpret water chemistry records preserved in shells and provide insights into biomineralization processes and bivalve biochemistry.

## Abstract 2

Barium and strontium isotopes in freshwater bivalve shells yield a record of past environments and provide important information about biogeochemical processes.

## 1 Introduction

The chemical and isotopic makeup of biogenic carbonates (e.g., bivalve shells, corals, otoliths, foraminifera, coccolithophores) is commonly used as an indicator of the environment in which they mineralized.^1−4^ However, the uptake of trace metals and the isotopic composition can be influenced by biological factors (sometimes referred to as “vital effects”) that complicate interpretations of carbonate chemistry,^5−9^ including species traits, the element or compound in question, mineralogy, calcification rate, food sources, and metabolic processes.^7,10−14^ Distinguishing and accounting for the role of biological factors in biogenic carbonate chemistry is paramount for accurately characterizing past environmental conditions.

Bivalves are largely sedentary animals that produce carbonate shells which often include annual layers, similar to tree rings.^15,16^ Most bivalves are filter feeders that capture suspended and dissolved material from the overlying water, from which it is assumed most food and material for shell production is obtained.^17−20^ Many species can live for decades, making their shells potentially valuable long-term recorders of the geochemical environment.^21,22^ Bivalves perform important ecosystem services in freshwater streams,^23−25^ which are among the world’s most threatened ecosystems and receive a wide range of pollutants from many sources.^26−30^ In addition, because of widespread river degradation, freshwater bivalves are among the most imperiled organisms on Earth.^23^ Therefore, geochemical information recorded in freshwater bivalve shells can be important for understanding both long-term changes in river environments and the causes of freshwater bivalve declines.

Marine bivalve shells have long been used as archives of geochemical proxies for a wide range of environmental conditions such as pollution, ocean water temperature, primary productivity, phytoplankton dynamics, and river discharge.^1,31−38^ However, freshwater environments are more geochemically and physically variable than marine environments. In contrast to ocean water, freshwater systems are also generally undersaturated with respect to calcium carbonate. This means that freshwater bivalves must concentrate ions to a greater extent than marine bivalves for carbonate shell mineralization to take place, potentially requiring different ion transport mechanisms. In addition, many marine bivalve shells have mixed mineralogy and include layers of aragonite and calcite (both CaCO_3_), while freshwater bivalves are made up almost entirely of aragonite.^39−42^

The alkaline earth metals barium (Ba) and strontium (Sr) and their isotopes are potentially informative proxies in freshwater systems. Stable Ba and radiogenic Sr isotopes in environmental samples provide insight into weathering and ecological processes^43−46^ and can be sensitive tracers of anthropogenic pollution from energy extraction and refining wastes (e.g., hydraulic fracturing produced water), with different waste sources having distinct Ba and Sr isotope signatures.^2,47−50^ Barium and Sr isotope variation in riverine samples are also important in constraining long-term climate-driven hydrologic and biogeochemical changes and can be used to understand the extent of river discharge to oceans and marine productivity.^51−53^ These elements substitute for Ca within the orthorhombic aragonite lattice of freshwater bivalve shells more readily than in the rhombohedral calcite lattice of marine shells.^54,55^

Radiogenic Sr isotopes, in which the isotope ^87^Sr is enriched to a variable extent by the long-term radioactive decay of ^87^Rb (half-life ≈49 billion years), provide a robust tool for identifying geologic and anthropogenic Sr sources, and the ^87^Sr/^86^Sr isotope ratio is affected only to a negligible extent by mass-dependent isotope fractionation during incorporation into carbonate. Therefore, it is expected that a precipitating shell will have the same ^87^Sr/^86^Sr as its source. In contrast, variation in the Ba isotope ratio (^138^Ba/^134^Ba) is caused entirely by mass fractionation during geologic, biologic, hydrologic, and anthropogenic processes. As the isotopes of Ba move through the environment and are mineralized into the shell, they may undergo some amount of fractionation that changes the ^138^Ba/^134^Ba ratio preserved in the shell.^56−60^ For shell Ba isotope signatures to be useful as a tracer of riverine chemistry or pollution, the extent and consistency of fractionation that occurs between the source and the shell must be known.

We examined the factors contributing to the Ba and Sr isotope composition of shells of six freshwater bivalve species, both native mussels and invasive species, at seven sites in the upper Ohio River basin. At each site, we collected shells and measured Ba and Sr isotope chemistry of river water over several months to capture seasonal variability. Our study species represented a wide range of phylogenetic groups and life history traits. We examined how species identity and growth rate at the time of shell deposition influenced isotope composition. Our primary objectives were to (1) determine the fidelity of shells in recording streamwater isotope composition; (2) understand how biological factors affect the fractionation of Ba isotopes between river water and shells, (3) evaluate how our findings inform the utility of bivalve shells as proxies of past environmental conditions in rivers, and (4) discuss how our findings inform an understanding of biomineralization processes and bivalve biochemistry.

## 2 Materials and Methods

### 2.1 Site Location and Field Methods

**Figure 1:**
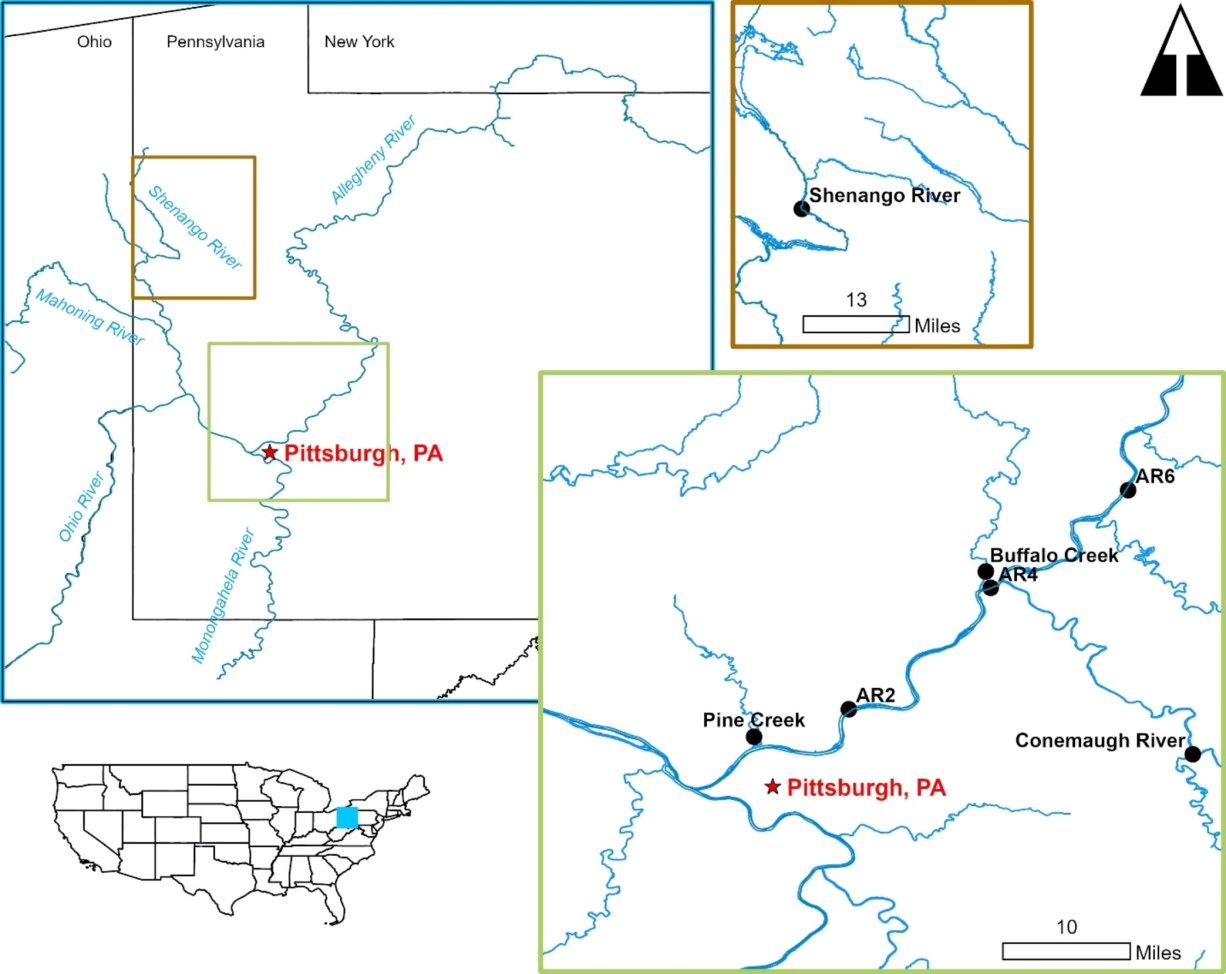
Map of the study area in western Pennsylvania (USA) showing sites of water and shell sample collection. River water samples and bivalve shells collected at each site identified by points. AR = Allegheny River; the number corresponds to the navigational pool.

River water samples were collected during 2021 and 2022 from seven sites in five streams in the upper Ohio River basin in western Pennsylvania (Figure 1, Table S1). In the lower section of the Allegheny River, which is impounded by a series of navigation dams, samples were collected from navigation pools 2, 4, and 6.^61^ Other sample locations included the Conemaugh River, Buffalo Creek, Shenango River, and Pine Creek. These four streams are unimpounded at our study sites, but the Shenango River and Conemaugh River are influenced by upstream reservoirs.

Because freshwater bivalves grow and mineralize shell primarily during warmer months (April-October),^62^ river water samples were collected primarily to coincide with that period. Water samples were collected approximately monthly at most sites to capture seasonal variability in water chemistry during the growing season. Only three samples were taken at the Shenango River, from July to December 2022, and a single sample was taken at Pine Creek, in October 2022. For each sampling event, approximately 250 mL of river water were collected 5–15 cm from the surface and field-filtered using 0.45-μm syringe filters (Whatman) into acid-cleaned high-density polyethylene sample bottles and preserved immediately to 2% HNO_3_ (Optima grade).

Bivalve shells were collected in 2022 from all sites. Shells were obtained from live bivalves collected by hand in the stream or from muskrat middens on shore; shells from muskrat middens had soft tissue remaining in the shells, indicating that they were harvested by muskrats recently, and therefore shell growth occurred during the period of water sample collection. Bivalve species were selected at each site based on availability (Table S2). The six study species represent a wide range of phylogenetic groups and life histories. Four native freshwater mussel species in three tribes in the order Unionida were collected (tribe Amblemini: *Amblema plicata*; tribe Lampsilini: *Potamilus alatus*, *Obliquaria reflexa*; tribe Pleurobemini: *Fusconaia flava*; all family Unionidae; hereafter, “unionids”). Two invasive species, *Corbicula fluminea* (order Venerida) and *Dreissena polymorpha* (order Myida), were also collected (Asian clam and zebra mussel, respectively). In general, growth rate is lowest and life span is greatest for *A. plicata* and *F. flava*, intermediate for *P. alatus* and *O. reflexa*, and growth rate is highest and life span is shortest for *C. fluminea* and *D. polymorpha*.^24^

### 2.2 Shell Sample Processing

**Figure 2:**
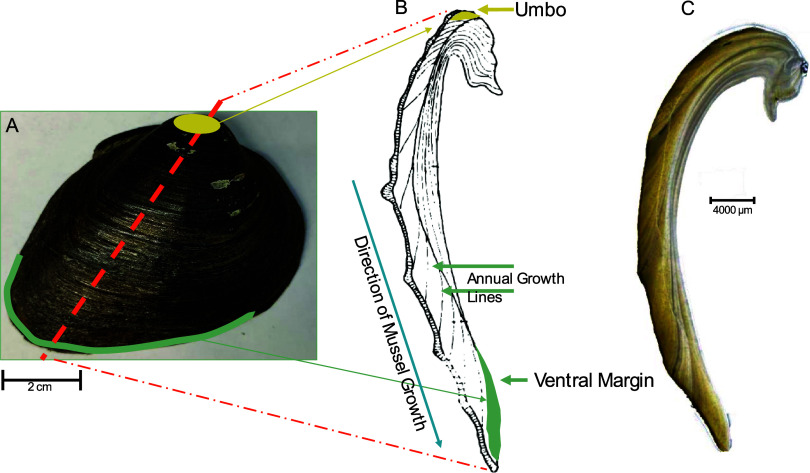
(A) *F. flava* shell illustrating where samples were extracted from ventral margin and umbo. Axis of maximum growth shown, where each shell was cut and thin-sectioned to count and measure annual growth lines. (B) Illustration of shell thin-section with annual growth lines identified, and locations of ventral margin and umbo. (C) Photograph of *F. flava* shell in thin-section, 7-years old.

For unionids, approximately 70 mg samples of shell material for chemical analysis were removed from each shell using a drill and 3 mm tungsten-carbide bit. The resulting powdered shell material was collected into clean glass vials. Bivalve shells grow radially, such that the outer, ventral margin of the shell represents the most recently mineralized material and the shell nearer the umbo represents material mineralized earlier in the animal’s life (Figure 2). Growth is most rapid in the first few years of life and declines subsequently;^24^ the age and growth rate at the time of mineralization for any location on the shell can be determined by interpreting annual rings. All the unionid shells we collected were adults for which growth rates at the time of collection were low. One sample was taken along the ventral margin from each of the 19 unionid specimens to represent shell mineralized during periods of slow growth at the time specimens were collected. Shell material was collected as close to the ventral margin as possible (within approximately 5 mm of the ventral margin) to represent the most recently mineralized material, while ensuring sufficient shell material for isotope analysis. This quantity of ventral margin shell material represented between one and 11 years, depending on the spacing of annual growth rings (see subsequent). For four unionid specimens (one *F. flava*, one *P. alatus*, and two *A. plicata*), samples were also collected from the umbo region to represent shell mineralized during periods of rapid growth early in the animal’s life. In total, this resulted in 19 ventral margin samples and four umbo samples.

We determined the age and growth rate associated with each unionid shell sample by interpreting annual rings in the shells (Figure 2, Tables S3 and S4). Radial thin sections (∼300 μm thickness) were prepared from one valve of each specimen using a low-speed saw with a diamond-impregnated blade (Buehler Ltd., Lake Bluff, IL), and were wet-sanded on a series of progressively finer sandpaper (400, 600, and 1500 grit) to ensure that growth lines were interpretable under a microscope. Thin sections were examined at 10× magnification under a binocular microscope and transmitted light, and annual rings were identified following criteria described by Haag and Commens-Carson.^16^ Imaging software was used to produce a composite photograph of each thin section, and for each photograph we measured the distance along the shell surface between each successive pair of annual rings to estimate growth increments produced in each year of the animal’s life. For each year of life, the increment width in that year and all previous years were summed to obtain shell height at that age. Instantaneous growth was calculated for each year as ln(*H*_t_/*H*_t-1_), where *H*_t_ is height at the end of that year and *H*_t-1_ is height at the end of the previous year.^63^ For the first year of life, we estimated *H*_t-1_ as the height of glochidia larvae for each species, obtained from Barnhart et al.^64^ (Tables S3 and S4).

Instantaneous growth during years of shell production that we sampled for isotope composition was estimated as follows. For ventral margin samples, the area from which shell material was sampled was marked on each thin section, and the number of years of shell production that made up this area was counted. In most specimens, we were unable to sample a single year at the ventral margin because low growth rates resulted in tightly crowded annual rings. Our ventral margin samples represented growth over a span of 1–11 years (mean = 3.5; Table S2). For umbo samples, the number of years sampled was determined based on examination of thin sections and annual rings on the shell surface. Because of rapid growth near the umbo, we were able to sample one or two years of shell production for all specimens (mean = 1.8). For both ventral margin and umbo samples, if more than one year was sampled, we used the mean instantaneous growth across all sampled years.

Because shells of *C. fluminea* and *D. polymorpha* were small (maximum length = 16 mm), we were unable to retrieve sufficient shell material for analysis from only the ventral margin or umbo. Consequently, shells of these species were crushed whole in a mortar and pestle. Growth rates of *C. fluminea* were estimated from the literature (Table S5).^65−72^ From these studies, shell height at age for *C. fluminea* was estimated from reported values or visual interpolation of length–frequency histograms. We calculated mean height at age across all studies and used those values to calculate instantaneous growth in each year of life as described for unionids (Table S6). We estimated all *C. fluminea* in our samples to be ≤2 years old, based on mean height at age from the literature (Table S2). Because we dissolved entire shells of *C. fluminea*, we estimated growth of all individuals as mean instantaneous growth over each estimated lifespan (see Table S2). We did not estimate growth for *D. polymorpha*.

Shell material was dissolved for further analysis by sequential extraction, ultimately yielding only the carbonate fraction of the shell. First, shell material was leached three times using ultrapure water (18.2 MΩ-cm at 25 °C) to remove lightly adsorbed phases. Shell material was then leached in a 25% hydrogen peroxide (H_2_O_2_) solution to extract and discard organic matter. The remaining carbonate was fully dissolved in 1 M acetic acid (∼12 h), centrifuged to separate out any remaining periostracum (the thin organic outer covering of the shell), and the supernatant was transferred to acid-washed PMP beakers. Dissolved shell material was evaporated to dryness at 90 °C and redissolved in 2% HNO_3_. Samples were centrifuged again to ensure removal of all solids and transferred via pipet to acid-cleaned 30 mL HDPE bottles. Dissolved shell samples were stored in 26 mL of 2% HNO_3_ for elemental and isotopic analysis. All reagents used were Optima grade.

### 2.3 Elemental and Isotope Analysis

All shell and water samples were first analyzed for elemental composition using ICP-MS at Northwestern University or University of Pittsburgh. For quality control, duplicates (at least one duplicate for every ten samples) and equipment blank samples were collected in the field during each sampling event. Duplicates for shell samples were split from the respective aliquot of dissolved shell powder. An internal standard of dissolved shell material was also analyzed with each batch of samples.

Samples for isotope analysis were prepared under Class 100 clean lab conditions. Samples were prepared for Ba isotope analysis following Tieman et al.^48^ and Matecha et al.^73^ Briefly, aliquots of each sample containing approximately 2 μg of Ba were spiked with a calibrated ^135^Ba–^137^Ba solution, evaporated to dryness, and redissolved in 0.5 mL of 2.0 N HCl. We used a ^135^Ba–^137^Ba double spike to correct for mass fractionation during chemical processing and mass spectrometry. This method also allowed precise determination of Ba concentrations in all samples. To separate Ba from the sample matrix, samples were loaded into disposable polypropylene gravity flow ion exchange columns containing Bio-Rad AG 50W-X8 200–400 mesh cation exchange resin conditioned in 2.0 N HCl and eluted with 2.5 N HCl followed by 2.0 N HNO_3_.^73^ This procedure is effective in separating Ba from major matrix elements as well as from La and Ce, which exhibit isobaric interferences with ^136^Ba and ^138^Ba.^73^

The eluted sample cut containing Sr was collected during the Ba column chemistry procedure. From this cut, Sr was further separated from the sample aliquot following the method of Wall et al.^74^ Aliquots containing approximately 2 μg Sr were purified by dissolving in 8 N HNO_3_ and loaded into disposable columns containing Eichrom Sr Resin. Matrix elements were removed with 8 N HNO_3_, and Sr was collected with ultrapure water.

Barium and Sr isotopes were measured on the DOE-NETL Neptune Plus multicollector ICP-MS at the University of Pittsburgh. For Ba isotopes, mass fractionation was corrected iteratively using the exponential law based the known isotopic composition of the ^135^Ba–^137^Ba double spike. A dissolved solution of the U.S. National Institute of Standards and Technology Standard Reference Material 3104a (NIST 3104a) spiked with the same ^135^Ba–^137^Ba mixture was measured throughout the analytical run to monitor any nonexponential instrument mass fractionation,^75^ and samples were normalized to the average standard value. In our study, δ^138^Ba is defined as the per mil deviation of the ^138^Ba/^134^Ba ratio from the NIST 3104a as
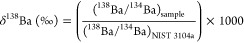
Error around estimates of δ^138^Ba reported in Tables S1 and S2 represent 2× the standard error of 50 measured ratios. For Sr, isotope mass fractionation during analysis was corrected using the exponential law with ^86^Sr/^88^Sr = 0.1194. Standard NIST SRM987 was monitored repeatedly during each analytical session, and the ^87^Sr/^86^Sr ratios of all samples are normalized such that SRM987 ^87^Sr/^86^Sr = 0.710240. The uncertainties in ^87^Sr/^86^Sr reported in Tables S1 and S2 represent 2 x the standard error of 60 measured ratios.

## 3 Results and Discussion

### 3.1 Strontium and ^87^Sr/^86^Sr Relationships between Shells and Water

**Figure 3:**
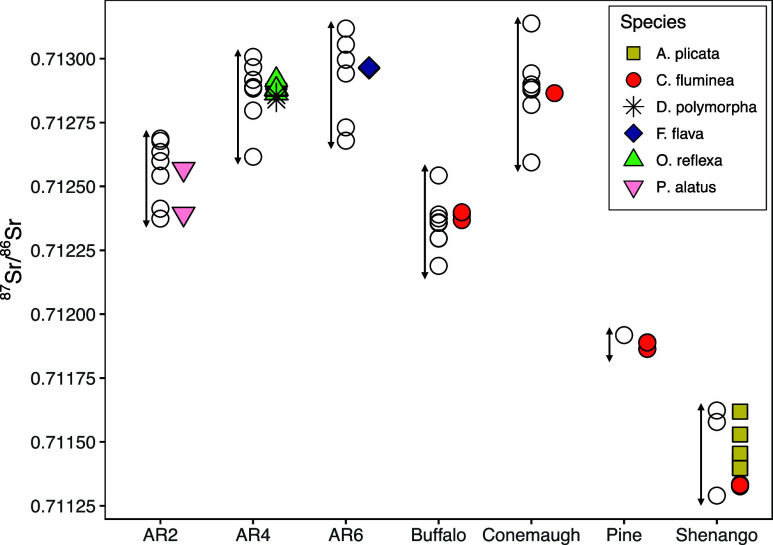
Sr isotope ratios for each sample collected, with stream samples (open circles) plotted with corresponding shells. ^87^Sr/^86^Sr values for all shell samples fall within the range of ^87^Sr/^86^Sr values for streamwater from which it was collected. Analytical error for each sample is smaller than symbol. AR = Allegheny River, with numbers corresponding to navigation pool.

River water samples at each site exhibited temporal variation in ^87^Sr/^86^Sr, greater than analytical error (Figure 3). However, the degree of temporal variation was low (<± 0.00055 for each site), and several sites had distinct ^87^Sr/^86^Sr signatures. For example, the range of variation in ^87^Sr/^86^Sr in the Shenango River overlapped with no other sites, and even within the Allegheny River, AR2 overlapped minimally with AR4 and AR6. The ^87^Sr/^86^Sr value for Pine Creek also does not overlap with the range of values for other streams in our study; however, because only one sample was collected from Pine Creek, seasonal variability for this location is unknown. Strontium concentrations

The ^87^Sr/^86^Sr value of river water ultimately reflects the source of Sr.,^44^ The relative contribution of various sources to a river system fluctuates over the course of a year depending on the amount and location of rainfall, anthropogenic discharges, and variable weathering within the drainage basin.^45,76,77^^87^Sr/^86^Sr ratios of source limestone and shale in the watershed have a range that can span from at least 0.708 to 0.733.^78^ Therefore, some amount of temporal variability in ^87^Sr/^86^Sr ratios would be expected in rivers,^79^ emphasizing the importance of collecting multiple samples over time to fully capture the range of ^87^Sr/^86^Sr values. Shell samples in this study represent an approximate average of the shell ^87^Sr/^86^Sr composition over the duration of mineralization, which ranged between 1 and 11 years in our samples (Table S2). Collectively, the ventral margin and umbo samples represent mineralization which occurred between 1990–2022. Although we do not have temporal overlapping river water data from 1990–2021 for direct comparison to shells mineralized during these years, the Sr isotope values of the shells suggest that the average relative contribution of Sr sources in these rivers has not changed considerably over the time spans represented by shell samples. Strontium isotope values for all shell samples, including those mineralized in 2022 and those mineralized in 1990, fall within the range of Sr isotope values of respective river water collected in 2021–2022 (Figure 3).

Only one previous study has examined ^87^Sr/^86^Sr signatures of unionid shells, which also found that shells were indistinguishable from signatures of their respective river water. However, that study was limited to three shells of two species.^1^ Our results greatly expand on these data by including seven additional freshwater species, both native and invasive, and, together, these results demonstrate that the ^87^Sr/^86^Sr of freshwater bivalve shells reflect the isotope composition of dissolved Sr in their home stream.

### 3.2 Ba Isotope Relationships between Shells and Water

The Ba isotope composition of river water reflects the geological and anthropogenic sources in the drainage basin, the extent of weathering and precipitation, and the contributing biological and physical processes that fractionate Ba isotopes as it moves from its source to the river.^43,80,81^ As was observed for ^87^Sr/^86^Sr, the Ba isotope composition of river water would be expected to vary temporally due to the variability of the relative contribution of various Ba sources. For all streams but one, the maximum in-stream variation in Ba isotopic composition was <0.11‰, which is near the average uncertainty of ±0.04‰. The exception is the Shenango River, which varied by 0.17‰. Average δ^138^Ba values for each stream ranged from +0.16 to +0.29‰, which is consistent with δ^138^Ba values reported elsewhere in freshwaters.^43,48,51,82^ Most stream waters showed significant overlap in δ^138^Ba with other streams in the region, suggesting similar geologic control on the Ba isotope composition. However, the average δ^138^Ba value of the Conemaugh River (+0.16‰) is lower than the average value for all other streams, indicating some spatial variability within the watershed.

Shell samples in this study represent an approximate average of the shell δ^138^Ba composition over a duration of 1 to 11 years (Table S2). Collectively, the ventral margin and umbo samples represent mineralization that occurred in the time frame of 1990–2022. An obvious challenge in this study is that we do not have temporal overlapping river water data from 1990–2021 for direct comparison. To evaluate the consistency of stream δ^138^Ba over time, we compared Ba isotope values of shell samples from the same species, same location, and with similar growth rates, but which varied in the number of years represented. Shell specimens #14 and #15 (both *F. flava* from AR6 with similar growth rates, Table S2) had overlapping Ba isotope values, and yet one represented mineralization during the years 2020–2022, and the other represented mineralization over a longer time span from 2015–2022. Specimens #35 and #37 (*A. plicata* from Shenango with similar growth rates) similarly had overlapping Ba isotope values, yet represented different time periods of 2014–2022 and 2017–2022, respectively. This lends support to the assumption that the isotopic composition of the source of Ba in these shells did not vary considerably across time spans represented by shell samples, allowing us to compare shell δ^138^Ba values to average river water values in 2021–2022. This is supported by the relative consistency of shell ^87^Sr/^86^Sr ratios over time in each of the streams sampled (Table S2).

**Figure 4:**
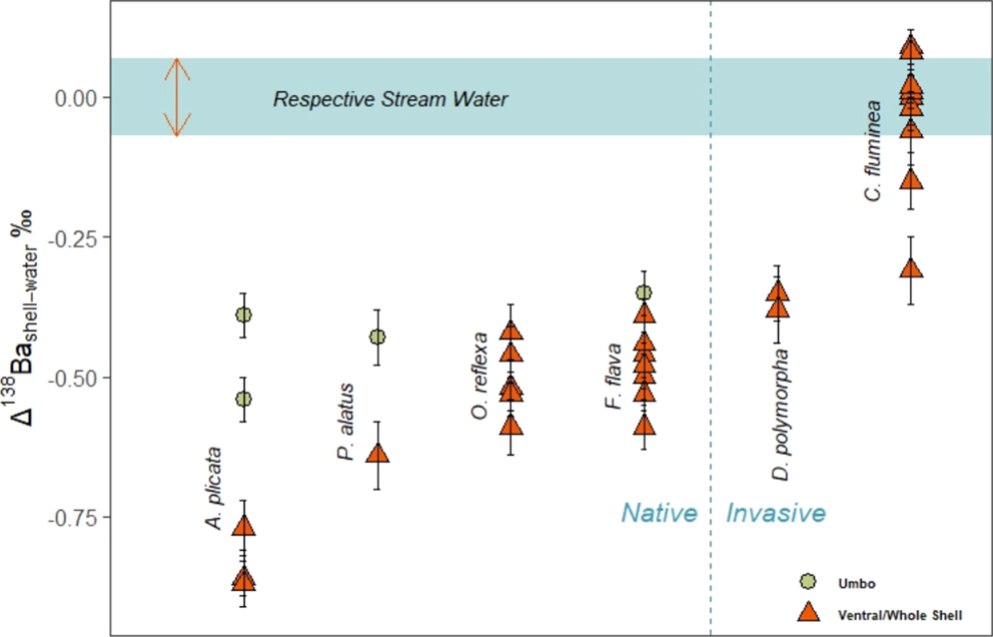
Ba isotopic offset (δ^138^Ba_shell_ – δ^138^Ba_water_) in shells of six freshwater bivalve species compared with the streamwater from which they were collected. The blue shaded region represents the maximum range of δ^138^Ba values measured from any individual stream in the study area. The average δ^138^Ba of each stream was normalized to Δ^138^Ba_shell-water_ = 0 so that the shell offsets could be directly compared.

Unlike ^87^Sr/^86^Sr, δ^138^Ba values of most shells departed widely from the δ^138^Ba values of river water in which they were growing (Figure 4). The δ^138^Ba values in shells varied across bivalve species and within individuals and ranged from −0.57 to +0.34‰. The offset of δ^138^Ba values in shells relative to river water (Δ^138^Ba_shell-water_) ranged from −0.86‰ to +0.09‰, with most shells enriched in lighter isotopes relative to river water. The only species for which δ^138^Ba values generally reflected that of river water was *C. fluminea* (mean Δ^138^Ba_shell-water_ = −0.03‰). The only exception to this is the single *C. fluminea* we collected from the Conemaugh River, for which the Δ^138^Ba_shell-water_ value was −0.31‰. The reason for this is unknown, but the Conemaugh River has large volume industrial wastewater treatment plants that discharge upstream of where the sample was collected.^47^ The large Ba isotope offset seen in this shell sample could result from isotopic variability in wastewater discharges upstream which were not captured by our monthly river water samples from this location.

The observed offset in δ^138^Ba between shells and river water suggest that *D. polymorpha* and unionids fractionate Ba isotopes during shell mineralization, resulting in enrichment in the lighter Ba isotope in shells relative to the Ba source. A similar effect was observed in inorganic and coral aragonite,^58,83,84^ which was attributed to kinetic effects (i.e., mineral growth rate) during aragonite precipitation.^59^ However, the magnitudes of offset reported here (up to −0.86‰) far exceed those reported for corals or any other biologically or inorganically precipitated aragonite, in which offset ranges only from −0.35 to +0.34‰,^58,59,85^ indicating that additional factors may be affecting isotope fractionation in freshwater bivalves.

### 3.3 Effects of Species and Growth Rate on δ^138^Ba Offset

The extent of the δ^138^Ba offset differed significantly among species (two-way ANOVA: F4,23 = 17.13, *P* < 0.0001). Growth rate also was a significant factor in explaining variation in δ^138^Ba offset (F1,23 = 216.44, *P* < 0.0001). Sums of squares indicated that more variation in δ^138^Ba offset was attributable to growth (1.68) than species (0.53). The species × growth interaction also was significant (F4,23 = 4.50, *P* = 0.0078), showing that the effect of growth is species-dependent. However, sums of squares indicated that little of the variation in δ^138^Ba offset was attributable to that interaction (0.14). Because of the significant interaction term, we did not attempt to examine pairwise differences among species. The ventral margin samples of *A. plicata* consistently had the greatest offset of any species (Δ^138^Ba_shell-water_ mean = −0.83‰), whereas shells of *C. fluminea* exhibited little or no offset from river water. These differences among freshwater species contrast with coral aragonite, where Ba isotope offset from ocean water is taxonomically invariant.^58,83^

**Figure 5:**
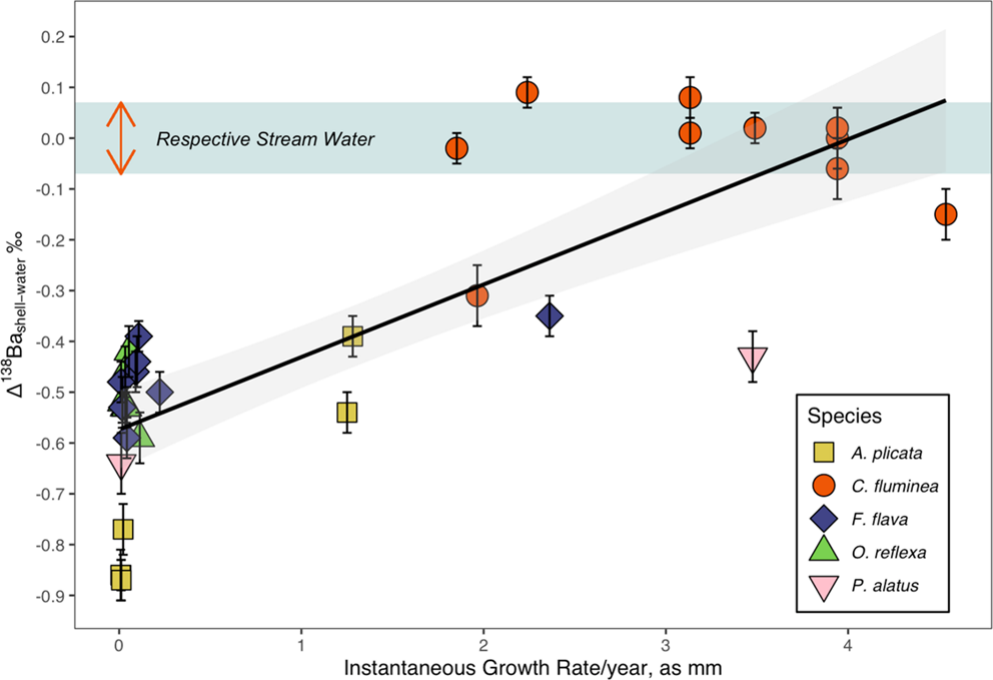
Ba isotopic offset from streamwater for all shell samples, plotted with respective instantaneous growth rates. Larger Ba isotopic offset exhibited by shells of slower growth. Linear regression shown with 95% confidence interval; δ^138^Ba offset = 0.145 × growth −0.576, *N* = 33, *P* < 0.0001. The blue shaded region represents the maximum range of δ^138^Ba values measured from any individual stream in the study area. The average δ ^138^Ba of each stream was normalized to Δ ^138^Ba_shell-water_ = 0 so that the shell offsets could be directly compared.

We evaluated the relationship between growth and δ^138^Ba offset for all shell samples (*D. polymorpha* excepted), ignoring species identity. This relationship explained 66.4% of the variation in δ^138^Ba offset (linear regression, δ^138^Ba offset = 0.145 × growth −0.576, *N* = 33, *P* < 0.0001; Figure 5). Along with the ANOVA results (see previous), this strong relationship suggests that growth rate is a more important factor than species in determining the extent to which Ba isotopes are fractionated as they are mineralized within the shells. Generally, shell samples representing slower growth rates had larger δ^138^Ba offset from river water (i.e., lighter Ba isotopes in the shell), and faster growing shells exhibited smaller offset. This relationship between growth rate and δ^138^Ba isotope offset was observed across species and was also apparent within individual shells.

For all three species for which we analyzed umbo samples (for which growth rate is higher), the δ^138^Ba offset was smaller than that of respective ventral margin samples (for which growth rate was lower). The umbo samples in our shells were mineralized between 1990 and 2008, and ventral margin samples were mineralized between 2011 and 2022. It is possible that differences in δ^138^Ba offset between umbo samples and respective ventral margin samples reflect differences in water chemistry over time. However, this explanation seems unlikely for several reasons. First, the four individuals were collected from two hydrologically distinct rivers (Shenango and Allegheny). If the Ba isotope chemistry of these rivers had changed between 1990–2008, any isotopic shifts would have affected both rivers similarly. Second, the umbo samples represent shell mineralized approximately during the years 2008, 2000, 1993, and 1990, and thus, any water chemistry change would have affected each of these years. This seems unlikely because of the large span of almost two decades. Finally, the ^87^Sr/^86^Sr value for each umbo sample falls within the range of ^87^Sr/^86^Sr values for stream samples collected in 2022 and showed no differences attributable to species or growth rate. In summary, growth rate appears to be an important factor in determining δ^138^Ba offset within individual shells.

Although isotopes were the focus of our study, we did observe some variation in elemental composition with species (Sr, Ba, and Ca elemental measurements included in Tables S1 and S1). Specifically, *D. polymorpha* shells tended to have higher Ba/Ca and Sr/Ca ratios than other species in our study. In comparing the *C. fluminea* shells with *A. plicata* shells from the same stream (Shenango), shells of *A. plicata* consistently had higher Ba/Ca ratios; however, the Sr/Ca ratios for both species collected from Shenango are similar. This supports our findings that there is species variability in the uptake and transport of these metals, but additional studies on this particular aspect of shell chemistry are needed.

### 3.4 Physical and Biological Factors Associated with Species and Growth Rate

Barium isotope fractionation may occur to some extent as the shell is mineralized from the extrapallial fluid. Enrichment of lighter isotopes is correlated with faster precipitation rates for alkaline earth metals in other carbonate minerals, such as Ca in calcite, Sr in aragonite, and Ba in witherite.^56,60,86−88^ In addition, the Ba isotope composition of inorganic aragonite precipitated under laboratory conditions depends on precipitation rate, where faster precipitation correlates with mineral enrichment in lighter isotopes.^59^ However, we observed *reduced* enrichment of light isotopes with increased growth rate (i.e., a positive relationship between Δ^138^Ba_shell-water_ and growth rate; Figure 5). If shell growth rates as measured in this study correspond to precipitation rates, then this relationship between growth rate and Ba isotope fractionation is in the opposite direction of offset observed in previous experimental studies and from theoretical expectations.^59^ Therefore, mineral precipitation kinetics are unlikely to be the sole cause of the δ^138^Ba offset observed in this study.

The presence of a transient intermediary amorphous calcium carbonate (ACC) phase could also affect the isotope ratios and concentrations of trace metals incorporated into shell material, causing them to deviate from expectations of a classic ion-by-ion aragonite crystallization pathway. Transient ACC phases have been noted in adult unionid shells,^89^ larval bivalve shells,^90^ as well as many other eukaryotic organisms with carbonate structures.^91,92^ ACC likely is formed in bivalves as the extrapallial fluid biomineralizes shell material. While the effects of an ACC phase on trace metal partitioning and isotopic fractionation into shell aragonite are unknown, Δ^138^Ba values for ACC formed by cyanobacteria deviate from fractionations expected in other carbonate minerals.^11^ An ACC phase could be partially responsible for the δ^138^Ba offset observed in unionid shells, and differences in the prevalence of ACC phases among species may explain some of the variation in Ba offset.

**Figure 6:**
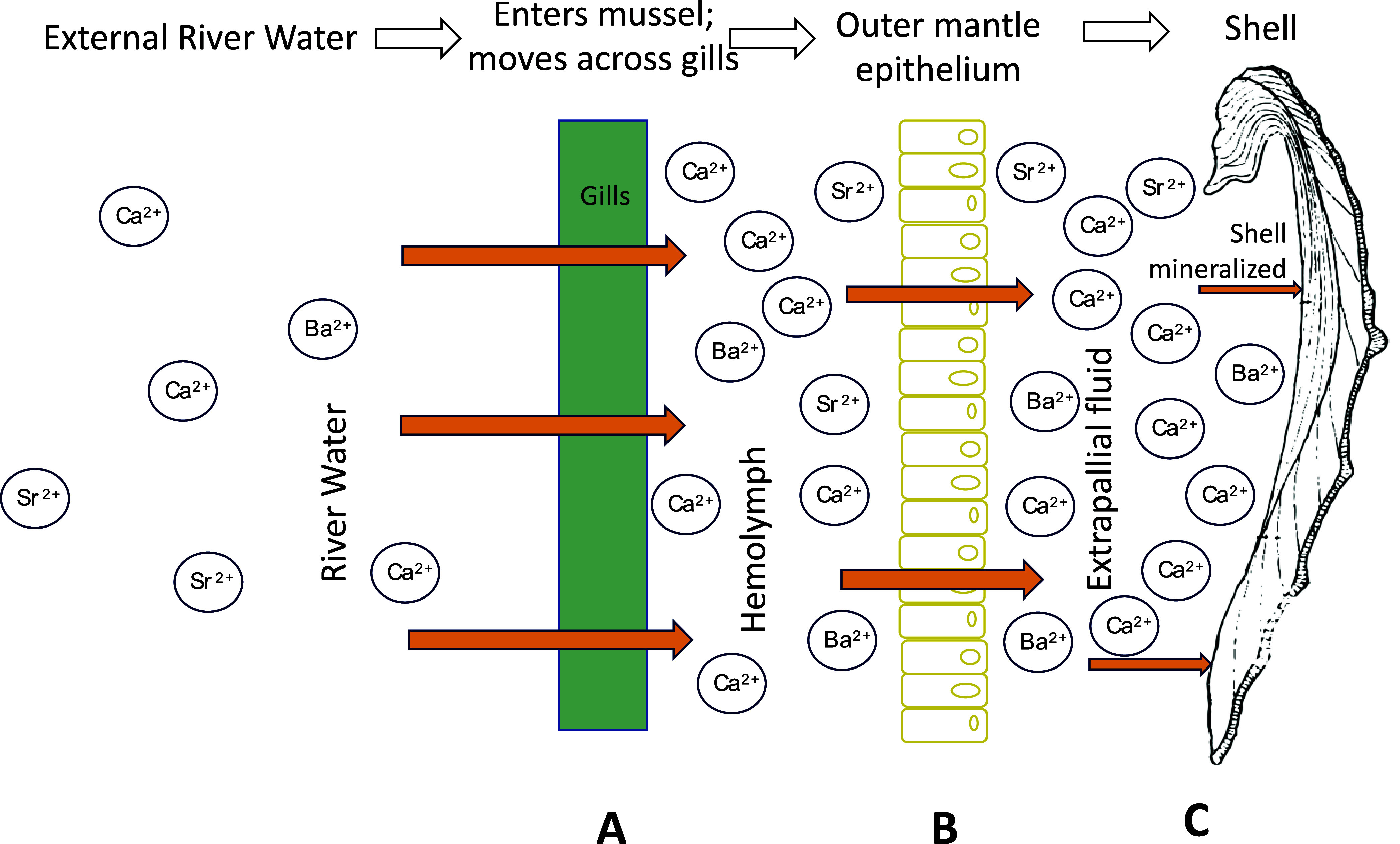
Schematic illustration showing ion movement from the external river water through the animal and into the shell. Three general interfaces across which Ba isotopes could undergo fractionation as they move through the animal: the gills and digestive tract, the mantle epithelium, and shell mineralization from the extrapallial fluid.

We suggest that ion transport processes within the animals are primarily responsible for the observed Ba isotope variations in shells. Barium and Sr are thought to behave as analogs of Ca through Ca ion transport pathways because they share chemical and physical properties.^10,93−96^ Bivalves take in ions from the river water across their gills as they filter feed, and they concentrate ions into the hemolymph (Figure 6A).^18^ In freshwater bivalves this step is likely attributed to energy-consuming active transport because the ion content of freshwater is much too low to support biochemical processes.^97,98^ Active transport rates would vary with metabolic/growth rates in the animal and could result in variable fractionation. Ions are also transported passively across the mantle epithelium via Ca-channels^99^ (Figure 6B), potentially incurring further isotope fractionation. As Ca-channels become saturated, ion selectivity is reduced thereby letting more non-Ca trace elements (e.g., Ba or Sr) through the channel.^100^ It is possible that reduction in ion selectivity resulting from channel saturation also results in reduced Ba isotope fractionation. How readily Ca-channels become saturated depends on channel density within the animal, and potentially growth rate or metabolic rate (i.e., faster growth/higher metabolism leading to greater likelihood of channel saturation as ions are transported more rapidly). Carré et al.^100^ further suggest that Ca-channel density in the mantle epithelium is likely species-dependent, which, if true, could also explain some of the apparent species-specific effects on Ba isotope fractionation. Importantly, whatever ion transport processes may fractionate Ba isotopes in freshwater bivalves, they do not occur to the same extent in *C. fluminea*.

### 3.5 Implications for Ba and Sr as Environmental Proxies

Strontium isotope ratios in shells faithfully and accurately reflected Sr isotope ratios in river water, regardless of bivalve species and growth rate, indicating that shells can provide useful and robust proxies for Sr isotope ratios in the environment over time. Because at least some streams have distinct Sr signatures, Sr isotopes also may be valuable for determining the provenance of historical shells in museum collections that lack accurate locality data. With the exception of *C. fluminea*, shells of freshwater bivalves had large Ba isotope offset relative to river water, and the magnitude of offset varied according to species and growth rate. Our results show that Ba records preserved in shells can be useful proxies if the magnitude of Ba isotope offset is known, allowing correction for the effects of species and growth. If the magnitude of offset is unknown, interpretational errors can be minimized by using the same species and specimens of similar age and growth rate when developing temporal chronologies of Ba isotope ratios. Moreover, our results provide insight into biomineralization processes and bivalve biochemistry. The lack of Ba isotope offset in *C. fluminea* suggests different ion transport pathways in that species, and the effects of species identity and growth rate on the magnitude of Ba isotope offset provide the basis for testable hypotheses about mechanisms of shell mineralization.

## Supporting Information Available

Concentrations, isotope ratios, and associated errors for all shell and water samples, growth increment measurements, and growth rate calculations included for all shell samples (PDF)
