# Supplementary material for: Controls on the Barium and Strontium Isotopic Records of Water Chemistry Preserved in Freshwater Bivalve Shells
Source: Environ Sci Technol. 2024 Aug 30;58(37):16454–64. doi: 10.1021/acs.est.4c05652 (PMC11411712; doi:10.1021/acs.est.4c05652)
Supplement: Supplementary file 1 — es4c05652_si_001.pdf [file es4c05652_si_001.pdf]

SUPPORTING INFORMATION

Controls on the barium and strontium isotopic records of water chemistry preserved in freshwater bivalve shells

Kristi S. Dobra[1]*, Rosemary C. Capo[1], Brian W. Stewart[1], Wendell R. Haag[2]

[1]Department of Geology and Environmental Science, University of Pittsburgh, Pittsburgh, PA 15260
[2]US Forest Service, Southern Research Station, Center for Bottomland Hardwoods Research, Frankfort, KY 40601
**Corresponding author; kristidobra@pitt.edu*

Summary: 13 pages, 6 tables.

Supporting information includes six tables which detail concentrations, isotope ratios, and associated errors for all shell and water samples. Growth increment measurements and growth rate calculations are also included for all shell samples.

Table S1: Barium isotope, Sr isotope, and Ba, Ca, and Sr concentration measurements for all river water samples. (Page S2)

Table S2: Barium isotope, Sr isotope, Ba/Ca, and Sr/Ca measurements for all shell samples. Also includes age of specimen, increment sampled, and instantaneous growth rate. (Page S3)

Table S3: Measurements of growth increments and length at age for all unionids. (Page S4-S10)

Table S4: Instantaneous growth calculations and age of shell increment sampled. (Page S11)

Table S5: Growth rates for *C. fluminea* obtained from literature. (Page S12)

Table S6: Growth rate calculations for *C. fluminea.* (Page S13)

SUPPLEMENTARY TABLE S1: RIVER WATER, ALL SAMPLES								
**Stream**	**Date (Month-Yr)**	**δ138Ba**	**Error (+/-)**	**$^{87}Sr/^{86}Sr$**	**Error (+/-)**	**Ba (ppb)**	**Sr (ppb)**	**Ca (ppb)**
Allegheny River- Pool 6	Apr 22	0.23	0.03	0.712996	0.000006	31.2	55.5	13,105
Allegheny River- Pool 6	May 22	0.24	0.03	0.712731	0.000013	33.9	60.6	15,897
Allegheny River- Pool 6	Jun 22	0.20	0.05	0.713055	0.000017	43.4	71.2	16,708
Allegheny River- Pool 6	Jul 22	0.27	0.06	-	-	39.6	103.2	19,125
Allegheny River- Pool 6	Aug 22	0.28	0.06	0.713118	0.000016	37.3	85.6	18,830
Allegheny River- Pool 6	Oct 22	0.30	0.03	0.712679	0.000021	37.8	89.4	17,888
Allegheny River- Pool 6	Dec 22	0.23	0.03	0.712942	0.000014	25.5	50.4	10,933
	**Average:**	**0.25**		**0.712920**				
Allegheny River- Pool 4	Apr 22	0.19	0.03	0.712967	0.000017	36.6	58.7	13,896
Allegheny River- Pool 4	May 22	0.30	0.04	0.712797	0.000015	33.0	62.1	16,194
Allegheny River- Pool 4	Jun 22	0.23	0.05	0.712883	0.000015	40.0	138.5	23,331
Allegheny River- Pool 4	Jul 22	0.24	0.05	0.712917	0.000018	43.9	157.8	25,682
Allegheny River- Pool 4	Aug 22	0.24	0.06	0.712889	0.000006	35.7	101.8	19,724
Allegheny River- Pool 4	Oct 22	0.28	0.02	0.712616	0.000007	34.9	60.8	17,246
Allegheny River- Pool 4	Dec 22	0.24	0.02	0.713007	0.000011	25.3	53.1	10,618
	**Average:**	**0.25**		**0.712868**				
Allegheny River- Pool 2	Apr 22	0.22	0.04	0.712374	0.000007	34.9	145.3	18,995
Allegheny River- Pool 2	May 22	0.26	0.03	0.712599	0.000007	36.1	99.0	18,212
Allegheny River- Pool 2	Jun 22	0.30	0.05	0.712635	0.000007	40.3	126.4	22,505
Allegheny River- Pool 2	Jul 22	0.31	0.05	0.712678	0.000013	78.3	166.0	25,402
Allegheny River- Pool 2	Aug 22	0.33	0.04	0.712687	0.000015	36.0	162.3	22,929
Allegheny River- Pool 2	Oct 22	0.29	0.02	0.712542	0.000016	35.0	114.8	22,310
Allegheny River- Pool 2	Dec 22	0.24	0.02	0.712413	0.000018	25.4	109.8	13,293
	**Average:**	**0.28**		**0.712561**				
Conemaugh River	Apr 22	0.16	0.04	0.712819	0.000015	34.9	97.3	19,539
Conemaugh River	May 22	0.16	0.06	0.712887	0.000007	42.0	168.3	33,722
Conemaugh River	Jun 22	0.13	0.04	0.712943	0.000019	48.8	289.0	44,429
Conemaugh River	Jul 22	0.16	0.05	0.712879	0.000015	46.4	247.7	47,782
Conemaugh River	Aug 22	0.16	0.07	0.712594	0.000014	41.4	217.7	32,522
Conemaugh River	Oct 22	0.20	0.06	0.713138	0.000013	40.7	296.1	51,201
Conemaugh River	Dec 22	0.15	0.04	0.712899	0.000018	32.0	137.7	22,702
	**Average:**	**0.16**		**0.712880**				
Buffalo Creek	Nov 21	0.27	0.06	0.712189	0.000014	66.9	226.0	39,900
Buffalo Creek	Apr 22	0.24	0.04	0.712543	0.000013	48.0	120.2	21,585
Buffalo Creek	May 22	0.27	0.03	0.712357	0.000007	52.0	285.9	40,533
Buffalo Creek	Jun 22	0.28	0.05	0.712297	0.000016	100.3	378.8	46,609
Buffalo Creek	Jul 22	0.17	0.08	0.712360	0.000014	92.1	311.6	47,837
Buffalo Creek	Aug 22	0.25	0.04	0.712296	0.000016	69.5	292.6	38,406
Buffalo Creek	Oct 22	0.24	0.03	0.712375	0.000012	72.2	216.1	38,042
Buffalo Creek	Dec 22	0.24	0.03	0.712390	0.000017	43.5	124.0	20,156
	**Average:**	**0.25**		**0.712351**				
Pine Creek	Nov 22	0.25	0.04	0.711918	0.000007	38.5	174.0	46,800
Shenango River	July 22	0.39	0.05	0.711290	0.000015	15.0	65.6	18,981
Shenango River	Oct 22	0.22	0.02	0.711623	0.000009	16.2	44.8	14,447
Shenango River	Dec 22	0.27	0.03	0.711578	0.000009	14.6	47.6	16,613
	**Average:**	**0.29**		**0.711497**				
*Only 1 sample collected from Pine Creek								
Error around estimates of δ138Ba reported in Tables S1 and S2 represent 2× the standard error of 50 measured ratios.								
The uncertainties in $^{87}Sr/^{86}Sr$ reported in Tables S1 and S2 represent 2 x the standard error of 60 measured ratios.								

SUPPLEMENTARY TABLE S2: SHELL DATA

Speciman ID #	Taxonomic Order	Species	Attribute	δ^{138}Ba	Error (+/-)	87Sr/86Sr	Error (+/-)	Age*	Age Sampled**	Instantaneous Growth Rate	Ba/Ca ppm (*1000)	Sr/Ca ppm (*1000)
Allegheny River- Pool 6												
15	Unionida	F. flava	Ventral Margin	-0.28	0.02	0.712962	0.000012	16	15-16	0.0169	0.26	0.84
14	Unionida	F. flava	Ventral Margin	-0.23	0.04	0.712956	0.000036	22	15-22	0.0106	0.24	0.72
14	Unionida	F. flava	Umbo	-0.1	0.04	0.712962	0.000017	22	1-2	2.362	0.24	0.84
Allegheny River- Pool 4												
1	Unionida	F. flava	Ventral Margin	-0.22	0.02	0.712880	0.000013	7	6-7	0.0903	0.35	0.98
2	Unionida	F. flava	Ventral Margin	-0.2	0.03	0.712901	0.000015	6	5-6	0.0984	0.13	1.33
3	Unionida	F. flava	Ventral Margin	-0.26	0.04	0.712893	0.000013	4	4	0.2227	0.11	1.10
4	Unionida	F. flava	Ventral Margin	-0.15	0.03	0.712888	0.000014	7	7	0.1074	0.13	1.38
5	Unionida	F. flava	Ventral Margin	-0.35	0.03	0.712882	0.000015	12	11-12	0.0425	0.22	0.82
6	Unionida	O. reflexa	Ventral Margin	-0.29	0.03	0.712865	0.000013	11	10-11	0.0427	0.31	0.67
7	Unionida	O. reflexa	Ventral Margin	-0.28	0.03	0.712886	0.000018	11	10-11	0.0326	0.23	1.03
8	Unionida	O. reflexa	Ventral Margin	-0.18	0.03	0.712879	0.000017	8	7-8	0.0536	0.20	1.00
9	Unionida	O. reflexa	Ventral Margin	-0.28	0.02	0.712881	0.000017	11	10-11	0.0178	0.32	0.74
10	Unionida	O. reflexa	Ventral Margin	-0.35	0.03	-	-	7	6-7	0.1133	0.29	0.78
11	Unionida	O. reflexa	Ventral Margin	-0.21	0.03	0.712917	0.000007	11	10-11	0.0331	0.19	0.90
12	Unionida	O. reflexa	Ventral Margin	-0.29	0.03	0.712879	0.000017	7	6-7	0.0395	0.20	0.79
41	Myida	D. polymorpha	Whole Shell	-0.12	0.03	0.712840	0.000015	Unknown	Unknown	NA	0.55	1.71
42	Myida	D. polymorpha	Whole Shell	-0.15	0.03	0.712857	0.000020	Unknown	Unknown	NA	0.58	1.65
Allegheny River- Pool 2												
13	Unionida	P. alatus	Umbo	-0.15	0.05	0.712393	0.000017	14	1	3.4764	0.28	1.45
13	Unionida	P. alatus	Ventral Margin	-0.36	0.06	0.712568	0.000025	14	9-14	0.0116	0.31	0.95
Conemaugh River												
38	Venerida	C. fluminea	Whole Shell	-0.15	0.03	0.712865	0.000007	1.8	1.8	1.9652	0.19	1.01
Buffalo Creek												
B1	Venerida	C. fluminea	Whole Shell	0.34	0.03	0.712388	0.000014	1.5	1.5	2.2395	0.24	1.03
B2	Venerida	C. fluminea	Whole Shell	0.27	0.04	0.712368	0.000015	1.0	1.0	3.9411	0.22	0.98
B3	Venerida	C. fluminea	Whole Shell	0.33	0.04	0.712399	0.000015	1.1	1.1	3.133	0.17	0.90
Pine Creek												
P1	Venerida	C. fluminea	Whole Shell	0.23	0.03	0.711864	0.000031	1.9	1.9	1.8528	0.11	1.03
P2	Venerida	C. fluminea	Whole Shell	0.26	0.03	0.711890	0.000016	1.1	1.1	3.133	0.11	1.06
P3	Venerida	C. fluminea	Whole Shell	0.27	0.03	0.711889	0.000012	1.0	1.0	3.4887	0.11	1.08
Shenango River												
36	Unionida	A. plicata	Umbo	-0.09	0.04	0.711529	0.000026	32	1-2	1.2818	0.13	0.54
36	Unionida	A. plicata	Ventral Margin	-0.56	0.05	0.711424	0.000027	32	22-32	0.008	0.27	0.45
37	Unionida	A. plicata	Umbo	-0.24	0.04	0.711619	0.000010	29	1-2	1.2509	0.18	0.59
37	Unionida	A. plicata	Ventral Margin	-0.56	0.03	0.711454	0.000005	29	22-29	0.0103	0.69	0.51
34	Unionida	A. plicata	Ventral Margin	-0.47	0.03	0.711398	0.000005	13	9-13	0.0219	0.26	0.51
35	Unionida	A. plicata	Ventral Margin	-0.57	0.04	0.711397	0.000010	17	13-17	0.0117	0.22	0.59
39	Venerida	C. fluminea	Whole Shell	0.24	0.04	0.711335	0.000007	1.0	1.0	3.9411	0.06	0.68
40	Venerida	C. fluminea	Whole Shell	0.15	0.03	0.711326	0.000005	0.9	0.9	4.5367	0.09	0.67
60	Venerida	C. fluminea	Whole Shell	0.3	0.06	0.711331	0.000006	1.0	1.0	3.9411	0.06	0.54

*Age of the mussel based on annual growth rings; C. fluminea growth rates were determined based on length data compiled from the literature

** Age range of the mussel that corresponds to the increment retrieved for isotopic analysis; for C. fluminea, entire shell was crushed for analysis.

Error around estimates of δ138Ba reported in Tables S1 and S2 represent 2× the standard error of 50 measured ratios.

The uncertainties in $^{87}Sr/^{86}Sr$ reported in Tables S1 and S2 represent 2 x the standard error of 60 measured ratios.

SUPPLEMENTARY TABLE S3: GROWTH INCREMENT MEASUREMENTS

Note: instantaneous growth for year 1 calculated by setting initial height as height of glochidia for each species as reported in Barnhart et al. (2008. JNABS 27:370-394)

Note: instantaneous growth estimates for individuals missing year 1 or 2 growth increments are average values for missing years and do not reflect year 1 growth

Species	specimen	age	Year	Increment height at age [µm]	Total height at age (um)	Instantaneous growth at age (/year)	Mean growth for samples
OREF	10	1	2016	7301.24	7301.24	3.5127	
OREF	10	2	2017	11436.08	18737.32	0.9425	
OREF	10	3	2018	6177.85	24915.17	0.2850	
OREF	10	4	2019	9752.12	34667.29	0.3303	
OREF	10	5	2020	7484.05	42151.34	0.1955	
OREF	10	6	2021	6696.81	48848.15	0.1475	
OREF	10	7	2022	4019.96	52868.11	0.0791	0.1133
OREF	6	1	2012	7317.59	7317.59	3.5149	
OREF	6	2	2013	7389.77	14707.36	0.6981	
OREF	6	3	2014	7888.23	22595.59	0.4294	
OREF	6	4	2015	11575.63	34171.22	0.4136	
OREF	6	5	2016	5411.6	39582.82	0.1470	
OREF	6	6	2017	6171.39	45754.21	0.1449	
OREF	6	7	2018	3411.96	49166.17	0.0719	
OREF	6	8	2019	3478.88	52645.05	0.0684	
OREF	6	9	2020	2248.01	54893.06	0.0418	
OREF	6	10	2021	2164.27	57057.33	0.0387	
OREF	6	11	2022	2723.74	59781.07	0.0466	0.0427
OREF	7	1	2012	6493.57	6493.57	3.3954	
OREF	7	2	2013	5951.11	12444.68	0.6505	
OREF	7	3	2014	8895.28	21339.96	0.5393	
OREF	7	4	2015	10358.93	31698.89	0.3957	
OREF	7	5	2016	6870.11	38569	0.1962	
OREF	7	6	2017	2739.53	41308.53	0.0686	
OREF	7	7	2018	5029.62	46338.15	0.1149	
OREF	7	8	2019	4073.05	50411.2	0.0842	
OREF	7	9	2020	3547.64	53958.84	0.0680	
OREF	7	10	2021	2176.28	56135.12	0.0395	
OREF	7	11	2022	1453.75	57588.87	0.0256	0.0326

OREF	8	1	2015	7024.98	7024.98	3.4741	
OREF	8	2	2016	9022.25	16047.23	0.8261	
OREF	8	3	2017	7634.2	23681.43	0.3892	
OREF	8	4	2018	4529.25	28210.68	0.1750	
OREF	8	5	2019	4751.71	32962.39	0.1557	
OREF	8	6	2020	2336.39	35298.78	0.0685	
OREF	8	7	2021	1729.5	37028.28	0.0478	
OREF	8	8	2022	2267.52	39295.8	0.0594	0.0536
OREF	12	1	2016	6969.81	6969.81	3.4662	
OREF	12	2	2017	10182.15	17151.96	0.9005	
OREF	12	3	2018	8786.03	25937.99	0.4136	
OREF	12	4	2019	7127.99	33065.98	0.2428	
OREF	12	5	2020	7535.48	40601.46	0.2053	
OREF	12	6	2021	1452.12	42053.58	0.0351	
OREF	12	7	2022	1884.85	43938.43	0.0438	0.0395
OREF	11	1	2012	4640.48	4640.48	3.0595	
OREF	11	2	2013	7125.84	11766.32	0.9304	
OREF	11	3	2014	9012.14	20778.46	0.5687	
OREF	11	4	2015	9015.84	29794.3	0.3604	
OREF	11	5	2016	5493.66	35287.96	0.1692	
OREF	11	6	2017	4150.97	39438.93	0.1112	
OREF	11	7	2018	3236.53	42675.46	0.0789	
OREF	11	8	2019	3509.75	46185.21	0.0790	
OREF	11	9	2020	3436.18	49621.39	0.0718	
OREF	11	10	2021	1301.06	50922.45	0.0259	
OREF	11	11	2022	2092.52	53014.97	0.0403	0.0331
OREF	9	1	2012	5952.53	5952.53	3.3085	
OREF	9	2	2013	8317.63	14270.16	0.8744	
OREF	9	3	2014	7871.47	22141.63	0.4393	
OREF	9	4	2015	9190.77	31332.4	0.3472	
OREF	9	5	2016	1648.58	32980.98	0.0513	
OREF	9	6	2017	3504.49	36485.47	0.1010	
OREF	9	7	2018	3977.26	40462.73	0.1035	
OREF	9	8	2019	2417.41	42880.14	0.0580	
OREF	9	9	2020	2180.22	45060.36	0.0496	
OREF	9	10	2021	741.73	45802.09	0.0163	
OREF	9	11	2022	889.09	46691.18	0.0192	0.0178
FFLA	1	1	2016	7509.82	7509.82	3.8805	

FFLA	1	2	2017	10253.97	17763.79	0.8610	
FFLA	1	3	2018	10064.77	27828.56	0.4489	
FFLA	1	4	2019	7318.79	35147.35	0.2335	
FFLA	1	5	2020	6777.87	41925.22	0.1763	
FFLA	1	6	2021	4214.07	46139.29	0.0958	
FFLA	1	7	2022	4089.34	50228.63	0.0849	0.0903
FFLA	2	1	2017	6762.71	6762.71	3.7758	
FFLA	2	2	2018	11056.63	17819.34	0.9689	
FFLA	2	3	2019	11836.41	29655.75	0.5094	
FFLA	2	4	2020	9957.28	39613.03	0.2895	
FFLA	2	5	2021	2981.23	42594.26	0.0726	
FFLA	2	6	2022	5635.88	48230.14	0.1243	0.0984
FFLA	3	1	2019	8948.77	8948.77	4.0558	
FFLA	3	2	2020	12699.28	21648.05	0.8834	
FFLA	3	3	2021	9499.41	31147.46	0.3638	
FFLA	3	4	2022	7768.94	38916.4	0.2227	0.2227
FFLA	4	1	2016	8056.8	8056.8	3.9508	
FFLA	4	2	2017	9079.88	17136.68	0.7547	
FFLA	4	3	2018	8331.49	25468.17	0.3962	
FFLA	4	4	2019	8274.23	33742.4	0.2813	
FFLA	4	5	2020	6730.44	40472.84	0.1819	
FFLA	4	6	2021	5848.77	46321.61	0.1350	
FFLA	4	7	2022	5253.14	51574.75	0.1074	0.1074
FFLA	5	2	2012	13508.47	13508.47		
FFLA	5	3	2013	9090.45	22598.92	0.5146	
FFLA	5	4	2014	8345.78	30944.7	0.3143	
FFLA	5	5	2015	5392.48	36337.18	0.1606	
FFLA	5	6	2016	3616.05	39953.23	0.0949	
FFLA	5	7	2017	2515.77	42469	0.0611	
FFLA	5	8	2018	1895.29	44364.29	0.0437	
FFLA	5	9	2019	1532.15	45896.44	0.0340	
FFLA	5	10	2020	1437.78	47334.22	0.0308	
FFLA	5	11	2021	1337.13	48671.35	0.0279	
FFLA	5	12	2022	2862.18	51533.53	0.0571	0.0425
FFLA	14	3		18287.98	18287.98		
FFLA	14	4		14036.07	32324.05	0.5696	
FFLA	14	5		6848.43	39172.48	0.1922	
FFLA	14	6		9347.94	48520.42	0.2140	

FFLA	14	7	4046.21	52566.63	0.0801	
FFLA	14	8	2282.76	54849.39	0.0425	
FFLA	14	9	2737.81	57587.2	0.0487	
FFLA	14	10	2383.15	59970.35	0.0405	
FFLA	14	11	781.33	60751.68	0.0129	
FFLA	14	12	766.66	61518.34	0.0125	
FFLA	14	13	641.24	62159.58	0.0104	
FFLA	14	14	1178.74	63338.32	0.0188	
FFLA	14	15	884.02	64222.34	0.0139	
FFLA	14	16	995.01	65217.35	0.0154	
FFLA	14	17	954.69	66172.04	0.0145	
FFLA	14	18	928.4	67100.44	0.0139	
FFLA	14	19	524.8	67625.24	0.0078	
FFLA	14	20	541.68	68166.92	0.0080	
FFLA	14	21	402.94	68569.86	0.0059	
FFLA	14	22	385.58	68955.44	0.0056	0.0106
FFLA	15	1	8644.23	8644.23	4.0212	
FFLA	15	2	5158.66	13802.89	0.4680	
FFLA	15	3	14823.3	28626.19	0.7294	
FFLA	15	4	7014.95	35641.14	0.2192	
FFLA	15	5	4242.9	39884.04	0.1125	
FFLA	15	6	5204.78	45088.82	0.1227	
FFLA	15	7	3768.25	48857.07	0.0803	
FFLA	15	8	2047.57	50904.64	0.0411	
FFLA	15	9	1860.72	52765.36	0.0359	
FFLA	15	10	2634.16	55399.52	0.0487	
FFLA	15	11	1227.66	56627.18	0.0219	
FFLA	15	12	947.65	57574.83	0.0166	
FFLA	15	13	1148.12	58722.95	0.0197	
FFLA	15	14	1224.65	59947.6	0.0206	
FFLA	15	15	862.44	60810.04	0.0143	
FFLA	15	16	1196.94	62006.98	0.0195	0.0169
FFLA	mean	1			3.9368	
FFLA	mean	2			0.7872	2.3620
PALA	13	1	12258.26	12258.26	3.4764	3.4764
PALA	13	2	13115.68	25373.94	0.7275	
PALA	13	3	14835.75	40209.69	0.4604	
PALA	13	4	16704.9	56914.59	0.3474	
PALA	13	5	9153.84	66068.43	0.1491	
PALA	13	6	3320.38	69388.81	0.0490	

PALA	13	7	2078.62	71467.43	0.0295	
PALA	13	8	1854.1	73321.53	0.0256	
PALA	13	9	2077.71	75399.24	0.0279	
PALA	13	10	745.55	76144.79	0.0098	
PALA	13	11	871.17	77015.96	0.0114	
PALA	13	12	597.18	77613.14	0.0077	
PALA	13	13	418.23	78031.37	0.0054	
PALA	13	14	562.58	78593.95	0.0072	0.0116
APLI	35	2	3851.83	3851.83	1.4601	1.4601
APLI	35	3	2189.09	6040.92	0.4500	
APLI	35	4	935.05	6975.97	0.1439	
APLI	35	5	1413.08	8389.05	0.1845	
APLI	35	6	1082.82	9471.87	0.1214	
APLI	35	7	690.48	10162.35	0.0704	
APLI	35	8	377.64	10539.99	0.0365	
APLI	35	9	271.63	10811.62	0.0254	
APLI	35	10	382.12	11193.74	0.0347	
APLI	35	11	174.58	11368.32	0.0155	
APLI	35	12	115.04	11483.36	0.0101	
APLI	35	13	208.98	11692.34	0.0180	
APLI	35	14	100.53	11792.87	0.0086	
APLI	35	15	156.2	11949.07	0.0132	
APLI	35	16	158.97	12108.04	0.0132	
APLI	35	17	64.58	12172.62	0.0053	0.0117
APLI	34	2	3312.99	3312.99	1.3848	1.3848
APLI	34	3	2465.2	5778.19	0.5562	
APLI	34	4	1988.11	7766.3	0.2957	
APLI	34	5	1653.85	9420.15	0.1931	
APLI	34	6	1123.78	10543.93	0.1127	
APLI	34	7	928.62	11472.55	0.0844	
APLI	34	8	877.02	12349.57	0.0737	
APLI	34	9	667.99	13017.56	0.0527	
APLI	34	10	230.4	13247.96	0.0175	
APLI	34	11	163.72	13411.68	0.0123	
APLI	34	12	186.86	13598.54	0.0138	
APLI	34	13	177.93	13776.47	0.0130	0.0219
APLI	36	2	2696.34	2696.34	1.2818	1.2818
APLI	36	3	1911.61	4607.95	0.5359	
APLI	36	4	1741.34	6349.29	0.3206	
APLI	36	5	1082.06	7431.35	0.1574	

APLI	36	6	1122.51	8553.86	0.1407	
APLI	36	7	903.1	9456.96	0.1004	
APLI	36	8	567.11	10024.07	0.0582	
APLI	36	9	373.84	10397.91	0.0366	
APLI	36	10	385.91	10783.82	0.0364	
APLI	36	11	251.27	11035.09	0.0230	
APLI	36	12	271.36	11306.45	0.0243	
APLI	36	13	171.69	11478.14	0.0151	
APLI	36	14	120.75	11598.89	0.0105	
APLI	36	15	140.46	11739.35	0.0120	
APLI	36	16	99.51	11838.86	0.0084	
APLI	36	17	89.71	11928.57	0.0075	
APLI	36	18	86.52	12015.09	0.0072	
APLI	36	19	74.29	12089.38	0.0062	
APLI	36	20	144.55	12233.93	0.0119	
APLI	36	21	200.31	12434.24	0.0162	
APLI	36	22	145.6	12579.84	0.0116	
APLI	36	23	202.16	12782	0.0159	
APLI	36	24	189.46	12971.46	0.0147	
APLI	36	25	157.58	13129.04	0.0121	
APLI	36	26	140.74	13269.78	0.0107	
APLI	36	27	54.87	13324.65	0.0041	
APLI	36	28	67.42	13392.07	0.0050	
APLI	36	29	42.68	13434.75	0.0032	
APLI	36	30	42.68	13477.43	0.0032	
APLI	36	31	54.28	13531.71	0.0040	
APLI	36	32	41.91	13573.62	0.0031	0.0080
APLI	37	2	2534.66	2534.66	1.2509	1.2509
APLI	37	3	1737.85	4272.51	0.5221	
APLI	37	4	997.53	5270.04	0.2098	
APLI	37	5	880.87	6150.91	0.1546	
APLI	37	6	752.34	6903.25	0.1154	
APLI	37	7	832.84	7736.09	0.1139	
APLI	37	8	446.61	8182.7	0.0561	
APLI	37	9	337.5	8520.2	0.0404	
APLI	37	10	561.16	9081.36	0.0638	
APLI	37	11	439.39	9520.75	0.0472	
APLI	37	12	258.98	9779.73	0.0268	
APLI	37	13	185.24	9964.97	0.0188	
APLI	37	14	148.68	10113.65	0.0148	
APLI	37	15	118.35	10232	0.0116	
APLI	37	16	123.25	10355.25	0.0120	

APLI	37	17	404.88	10760.13	0.0384	
APLI	37	18	195.45	10955.58	0.0180	
APLI	37	19	228.7	11184.28	0.0207	
APLI	37	20	213.65	11397.93	0.0189	
APLI	37	21	256.66	11654.59	0.0223	
APLI	37	22	281.05	11935.64	0.0238	
APLI	37	23	242.6	12178.24	0.0201	
APLI	37	24	212.91	12391.15	0.0173	
APLI	37	25	66.9	12458.05	0.0054	
APLI	37	26	53.57	12511.62	0.0043	
APLI	37	27	63.45	12575.07	0.0051	
APLI	37	28	38.71	12613.78	0.0031	
APLI	37	29	38.35	12652.13	0.0030	0.0103

SUPPLEMENTARY TABLE S4: INSTANTANEOUS GROWTH & AGE DATA										
Note: columns G and H provide estimates of mean growth during the years from which we sampled shell material, as indicated in columns E and F.										
Note: The FFLA specimen from which we the umbo didn't have age 1 and 2 growth estimates. I estimated mean growth for that sample based on the mean values of observed 1 and 2 year growth from other specimens for that species; see Increments tab										
Species	Specimen	Length (mm)	Age (y)	Ages sampled ventral	Ages sampled umbo	Mean instantaneous growth, ventral sample	Mean instantaneous growth, umbo sample			
OREF	6	53.3	11	10-11	-	0.0427	-			
OREF	7	56.2	11	10-11	-	0.0326	-			
OREF	8	37.3	8	7-8	-	0.0536	-			
OREF	9	42.7	11	10-11	-	0.0178	-			
OREF	10	50.5	7	6-7	-	0.1133	-			
OREF	11	44.6	11	10-11	-	0.0331	-			
OREF	12	42.8	7	6-7	-	0.0395	-			
FFLA	1	41.7	7	6-7	-	0.0903	-			
FFLA	2	43.6	6	5-6	-	0.0984	-			
FFLA	3	34.5	4	4	-	0.2227	-			
FFLA	4	41.6	7	7	-	0.1074	-			
FFLA	5	48.8	12	11-12	-	0.0425	-			
FFLA	14	75	22	15-22	1-2	0.0106	2.3620			
FFLA	15	59.6	16	15-16	-	0.0169	-			
PALA	13	133.1	14	9-14	1	0.0116	3.4764			
APLI	34	103.1	13	9-13	1-2	0.0219	1.3848			
APLI	35	101.5	17	13-17	1-2	0.0117	1.4601			
APLI	36	112.8	32	22-32	1-2	0.0080	1.2818			
APLI	37	118.1	29	22-29	1-2	0.0103	1.2509			

SUPPLEMENTARY TABLE S5: C. FLUMINEA GROWTH VALUES

Age	min length (mm)	max length (mm)	median length year 1	median length year 2		median length (mm)
Sickel (1977)--median lengths at age visually interpolated from Fig. 2						
1						8
2						16
3						24
Stites et al. (1995)--median lengths at age visually interpolated from Fig. 2						
Note: I used interpolated values instead of values in Table 2 because it was unclear what those values referred						
1			11	12.5		11.75
2			15	18.5		16.75
3			21			21
Henricksen and Bollens (2022)--used midpoint of ranges of length at age provided in last paragraph, page 50						
Note: seems like when they say "one-year old", they actually mean age 2						
Note: length of recruits stated as "less than 15 mm"; no value used for this study						
1						
2	13	25				19
3	20	33				26.5
4	30	43				36.5
Cataldo and Boltovskoy (1999)--median lengths at age visually interpolated from Fig. 4						
Note: I used interpolated values instead of the length ranges provided in the abstract because it was unclear what those referred to.						
1						10
2						20
3						25
4						29
5						30
Hornbach (1982)--median lengths at age visually interpolated from Fig. 1						
Note: I used interpolated values instead of values states in abstract because the former represent actual size at the end of each year						
1			2.5	2		2.25
2			11.1	11.1		11.1
3				17.6		17.6
Morton (1977)--median lengths at age visually interpolated from Fig. 7						
1						7.5
2						14.5
3						20
Li et al. (2017)						
Note: Values from Table 1. Used observed SHn for year 1, and corrected SHn for all other years; age 6 omitted because no other values from other studies						
1						11.1
2						16.25
3						19.95
4						22.13
5						25.56
Mouthon (2001)--values from Table 3						
	Sites					
1	7.5	2.2	4	6.2	1.65	4.31
2	12.5	11.4	10.8	13.5		12.05
3	19.4	18.5	20.5			19.46667
4	26.6	26.2	21.5			24.76667
5	25.6	27.3	29.2			27.36667
Summary			Age			
	1	2	3	4	5	
Sickel	8	16	24			
Stites	11.75	16.75	21			
Henricksen		19	26.5	36.5		
Cataldo	10	20	25	29	30	
Hornbach	2.25	11.1	17.6			
Morton	7.5	14.5	20			
Li	11.1	16.25	19.95	22.13	25.56	
Mouthon	4.31	12.05	19.47	24.77	27.37	
mean	7.84	15.71	21.69	28.10	27.64	
	8.433333	16.22857	22.00714	29.21	27.78	

SUPPLEMENTARY TABLE S6: C. FLUMINEA GROWTH CALCULATIONS						
Note: size at age 0 based on size of veliger as reported by McMahon (1991)						
von Bertalanffy parameters:						
k	0.4025					
Linf	34.048					
t0	0.3763					
Age	mean observed length (mm)	predicted length (mm)	Instantaneous growth at age (/year)	mean lifetime instant. Growth	increment (um)	mean increment (um)
0		0.25				
1	7.84	7.56	3.409024537	3.409025	7558.93	7558.93
2	15.71	16.34	0.770652288	2.089838	8777.25	8168.09
3	21.69	22.21	0.306937655	1.495538	5868.88	7401.69
4	28.10	26.13	0.162735658	1.162338	3924.20	6532.32
5	27.64	28.75	0.095692029	0.949008	2623.90	5750.63
SAMPLES			pred mean lifetime	pred age (yr)	mean increment (um)	
Identifier #	river	length (mm)	inst. Growth			
38	CON	15	1.9652	1.764	7732.20	
39	shen	7	3.9411	0.968	8220.92	
40	shen	6	4.5367	0.898	8265.32	
60	shen	7	3.9411	0.968	8220.92	
B-1	buff	13	2.2395	1.519	7879.89	
B-2	buff	7	3.9411	0.968	8220.92	
B-3	buff	9	3.1330	1.125	8122.33	
P-1	pine	16	1.8528	1.902	7650.83	
P-2	pine	9	3.1330	1.125	8122.33	
P-3	pine	8	3.4887	1.044	8173.32	
